# Combating Cariogenic *Streptococcus mutans* Biofilm Formation and Disruption with Coumaric Acid on Dentin Surface

**DOI:** 10.3390/molecules29020397

**Published:** 2024-01-13

**Authors:** Syed Sohail Ahmad, Muhammad Faisal Siddiqui, Farhana Maqbool, Ihsan Ullah, Fazal Adnan, Aqel Albutti, Noorah Alsowayeh, Ziaur Rahman

**Affiliations:** 1 Department of Microbiology, Hazara University, Mansehra 21300, Pakistan; syedsohail.edu@gmail.com (S.S.A.); fairy_es11@yahoo.com (F.M.); 2 Department of Biological Sciences, Faculty of Science, King Abdulaziz University, Jeddah 21589, Saudi Arabia; iullah@kau.edu.sa; 3 Atta Ur Rahman School of Applied Biosciences, National University of Sciences & Technology, Islamabad 44000, Pakistan; fazliadnan@gmail.com; 4 Department of Medical Biotechnology, College of Applied Medical Sciences, Qassim University, Buraydah 51452, Saudi Arabia; 5 Department of Biology, College of Science in Al-Zulfi, Majmaah University, Al-Majmaah 11952, Saudi Arabia; n.alsowayeh@mu.edu.sa; 6 Department of Microbiology, Abdul Wali Khan University, Mardan 23200, Pakistan; zrahman@awkum.edu.pk

**Keywords:** *Streptococcus mutans*, dental plaque, biofilm, coumaric acid, EPS, 3D plots, molecular interaction

## Abstract

*Streptococcus mutans*, the primary cause of dental caries, relies on its ability to create and sustain a biofilm (dental plaque) for survival and pathogenicity in the oral cavity. This study was focused on the antimicrobial biofilm formation control and biofilm dispersal potential of Coumaric acid (CA) against *Streptococcus mutans* on the dentin surface. The biofilm was analyzed by 3-(4,5-dimethylthiazol-2-yl)-2,5-diphenyl-2H-tetrazolium bromide (MTT) viability assay, microtiter plate assay, production of extracellular polymeric substances (EPSs), florescence microscopy (surface coverage and biomass μm^2^) and three-dimensional (3D) surface plots. It was observed that CA at 0.01 mg/mL reduced bacterial growth by 5.51%, whereases at 1 mg/mL, a significant (*p* < 0.05) reduction (98.37%) was observed. However, at 1 mg/mL of CA, a 95.48% biofilm formation reduction was achieved, while a 73.45% biofilm dispersal (after 24 h. treatment) was achieved against the preformed biofilm. The MTT assay showed that at 1 mg/mL of CA, the viability of bacteria in the biofilm was markedly (*p* < 0.05) reduced to 73.44%. Moreover, polysaccharide (EPS) was reduced to 24.80 μg/mL and protein (EPS) to 41.47 μg/mL. ImageJ software (version 1.54 g) was used to process florescence images, and it was observed that the biofilm mass was reduced to 213 (μm^2^); the surface coverage was reduced to 0.079%. Furthermore, the 3D surface plots showed that the untreated biofilm was highly dense, with more fibril-like projections. Additionally, molecular docking predicted a possible interaction pattern of CA (ligand) with the receptor Competence Stimulating Peptide (UA159sp, PDB ID: 2I2J). Our findings suggest that CA has antibacterial and biofilm control efficacy against *S. mutans* associated with dental plaque under tested conditions.

## 1. Introduction

Dental plaque is a soft, adhesive layer that develops on the outer surface of teeth. Dental plaque is also stated as a biofilm, which chiefly comprises bacteria and their byproducts, as well as water and the extracellular polymeric substances (EPS) produced. A biofilm is the result of a series of sequential processes, such as the formation of conditioning layer, initial attachment of microbes and plaque growth [1].

Dental plaque is a major oral disease of the teeth, which is caused due to various food materials, microbes, and their products [2,3]. In dental plaque, different microbes encase themselves in extracellular matrix [4]. *Streptococcus mutans* is mostly present in all dental plaque samples [3]. However, it is now known that the dental plaque consists of different and wide ranges of microbial species [5].

*S. mutans* regulates its biofilm through a density-dependent quorum sensing (QS) system, chiefly involving the Competence Stimulating Peptide (CSP) and the ComD/ComE two-component signal transduction system, constituting a significant aspect of its behavior. In addition to biofilm formation, the CSP-mediated QS system in *S. mutans* also plays a role in controlling acidogenicity, genetic transformation and bacteriocin production. Particularly, these traits are most effectively expressed in cells originating from a biofilm. Therefore, efforts are underway to target the *S. mutans*’ QS system to diminish biofilm formation and/or virulence, with the aim of developing therapeutic or preventive strategies against dental caries [6].

Bacteria in dental plaques have shown resistance to antimicrobial agents present in toothpaste and mouthwash. About 30% of chlorhexidine remains in the mouth from chlorhexidine doses and reveals good effectivity. It was found that chlorhexidine has strong microbial control potential against different microorganisms [7]. However, many microorganisms have shown resistance against chlorhexidine, and various factors have a particular impact on the resistance of biofilms [8]. It is known that many chemicals have no strong microbial biofilm control potential, and many microbes have shown resistance against different chemicals. Bacterial biofilms provide resistance to antibiotics and increased invasiveness capabilities. Several efforts have been undertaken to find new classes of small molecules that possess biofilm inhibitory activity against clinically relevant Gram-positive and Gram-negative pathogens [9,10].

Recently, many plant-based compounds have been employed to control dental plaque biofilms. These compounds have many benefits compared to synthetic chemicals [11]. Using plant extracts and compounds in dentistry provides an affordable option for many patients globally, addressing the global inequality in preventive dental care and the accessibility issues with fluoride for preventing dental caries [12]. Plant extracts with antimicrobial properties serve as broad-spectrum antibiotics, minimizing resistance development, inhibiting microbial growth, reducing virulence factors and exhibiting antibiofilm activity [13].

Various plant products were tested to control microbial biofilms [3,14,15]. Among plant-based compounds, coumaric acid (CA) is one of the compounds; it is derivative of cinnamic acid. CA is found in plants, such as beans, peanuts, basil and tomatoes. CA was found to have anti-bacterial potential against *Listeria monocytogenes* [16]. In a previous study, CA showed antimicrobial potential [17]. In another study, by Lou et al. [18], it was observed that p-coumaric acid effectively inhibited the growth of all test bacterial pathogens, and the MIC values ranged from 10 to 80 μg/mL CA. Furthermore, it was observed that CA at 0.2 mg/mL showed active growth inhibition against *Alicyclobacillus acidoterrestris* [19].

Dental plaque is difficult to control due to the resilience and rapid growth of certain microbes. It is known that chemical agents do not seem to have a clear-cut ability to efficiently mitigate microbial biofilms associated with dental plaque; in fact, these agents have shown resistance against dental plaque bacteria. Also, the use of such chemicals is linked with numerous adverse effects, such as teeth discoloration, gastrointestinal problems like diarrhea and the possibility to induce vomiting. Studies exhibited that many plants can produce compounds, which have the ability to control microbial biofilms [20].

This study was focused on the antimicrobial, biofilm formation control and biofilm dispersal potential of coumaric acid (CA) against *Streptococcus mutans* associated with dental plaque on the dentin surface. The biofilm was analyzed using an MTT viability assay, microtiter plate assay, production of extracellular polymeric substances (EPS), florescence microscopy (surface coverage and biomass μm^2^) and three-dimensional (3D) surface plots. Additionally, molecular docking was performed for possible interaction patterns of CA with Competence Stimulating Peptide (UA159sp, PDB ID: 2I2J) of *S. mutans.*

## 2. Results and Discussion

### 2.1. The Effect of CA on the Plonktonic Growth of Streptococcus mutans

**Figure 1:**
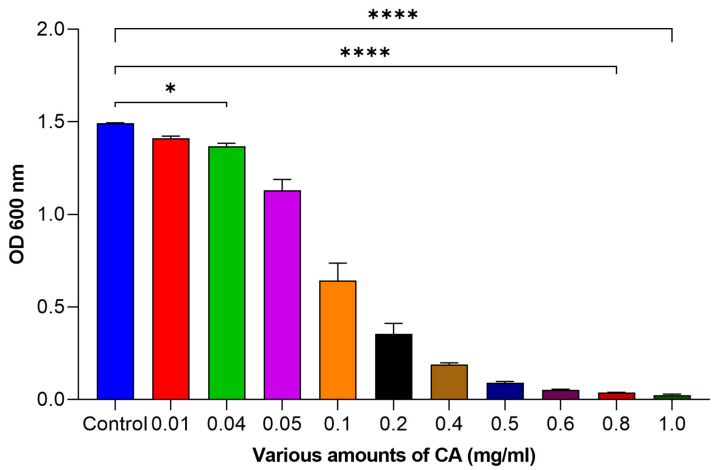
Influence of various amounts of CA on bacterial growth. The statistical tests used in these analyses were one-way ANNOVA followed by Tukey’s Multiple Comparisons Test. * *p* ≤ 0.05, **** *p* ≤ 0.0001.

It was revealed that using a small quantity of coumaric acid (CA) had only a slight impact on reducing bacterial growth. However, as CA was increased, more significant effects were observed on growth reduction (Figure 1). At 0.01 mg/mL of CA, we saw a 5.51% reduction and, at 0.2 mg/L, a 7.20% reduction. The effect became even more noticeable at higher amounts: 87.25% reduction at 0.4 mg/mL, 93.95% at 0.5 mg/mL and a remarkable 98.37% decrease at 1 mg/mL against *Streptococcus mutans*. These results highlight the potential of CA as a promising agent for bacterial growth reduction.

### 2.2. Effect of CA on Control of Biofilm Formation against Streptococcus mutans

**Figure 2:**
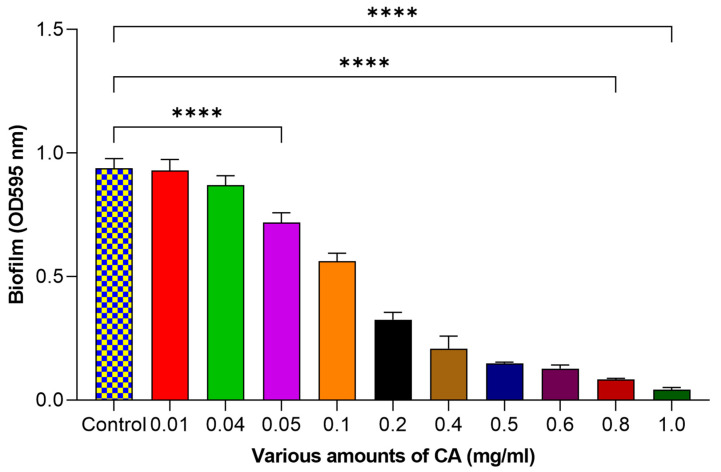
Influence of various amounts of CA on biofilm of *Streptococcus mutans.* The statistical tests used in these analyses were one-way ANNOVA followed by Tukey’s Multiple Comparisons Test. **** *p* ≤ 0.0001.

The results exhibited a clear and dose-dependent association between CA concentration and the mitigation of biofilm formation (Figure 2). At the lowest amount of CA tested (0.01 mg/mL), we observed a 0.87% reduction in biofilm formation. However, the impact became more evident as we raised the CA concentration. At 0.4 mg/mL, we noted a 7.10% reduction, and, at 0.5 mg/mL, an even more considerable reduction of 23.31% was observed. This trend continued as the concentration of CA increased to 0.1 mg/mL (40.05% reduction), 0.2 mg/mL (65.28% reduction) and 0.4 mg/mL (77.75% reduction). The efficacy of CA as a biofilm control agent remained consistent and increased significantly as we further increased the concentration to 0.5 mg/mL, where we achieved an 84.04% reduction. Notably, at 0.6 mg/mL, the results were particularly noteworthy, with an 86.35% reduction in biofilm formation. The effect continued to rise at 0.8 mg/mL, resulting in a significant 91.00% reduction. The most striking outcome was observed at the highest concentration of 1 mg/mL, where a 95.48% (*p* < 0.05) reduction in the biofilm formation was achieved. These outputs indicated that higher CA amounts are more effective in mitigating biofilm formation, highlighting the efficacy of CA as a potent agent for mitigating biofilms. In one of the previous studies, CA was used to control the *Salmonella enteritidis* biofilm. They used a small concentration of CA (0.5 mg/mL) to control biofilm formation [17].

### 2.3. Influence of CA on Bacterial Cell Viability

**Figure 3:**
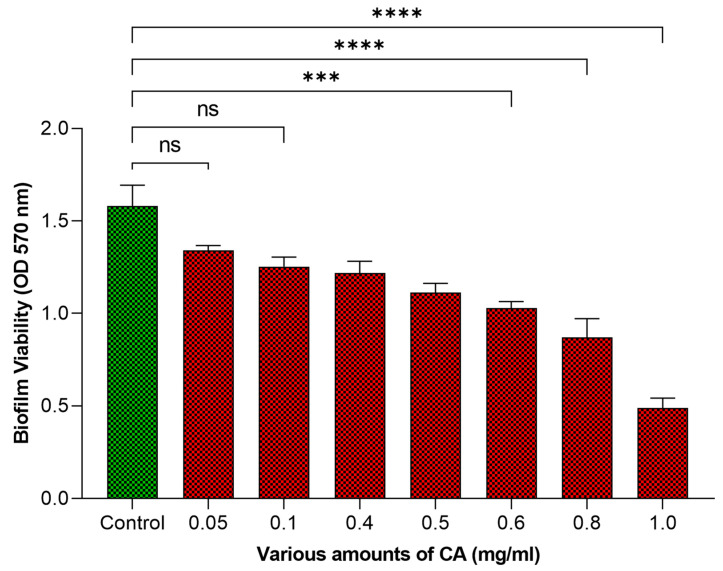
The effect of various amounts of CA on viability of bacterial cells in biofilm. The statistical tests used in these analyses were one-way ANNOVA followed by Tukey’s Multiple Comparisons Test. ns: non-significant, *** *p* ≤ 0.001, **** *p* ≤ 0.0001.

Our research results using the MTT assay to evaluate the effect of various amounts of CA on biofilm-associated bacteria revealed the antibacterial efficacy of CA. It was exhibited that CA can reduce bacterial viability within biofilms, marking a significant reduction in viable cells. This method not only exhibits a method for evaluating the antibacterial activity of CA against *Streptococcus mutans* biofilms but also features its substantial potential in biofilm control. At lower concentrations of CA (0.05–0.1 mg/mL), the reduction in viability was not significant (ns). Our results in Figure 3 exhibit that at 1 mg/mL, the bacterial viability in biofilms was reduced by 73.44%. These results hold great promise for the development of CA-based treatments aimed at combatting biofilm-associated bacterial infections and contribute to the broader understanding of biofilm control strategies. The MTT assay is used for bacterial cell viability. However, the MTT assay must be confirmed in some situations for cell viability in biofilms [21]. Previously, this technique has been used for viability in biofilms [22,23].

### 2.4. Influence of CA on Eradication of Preformed Biofilm of Streptococcus mutans

**Figure 4:**
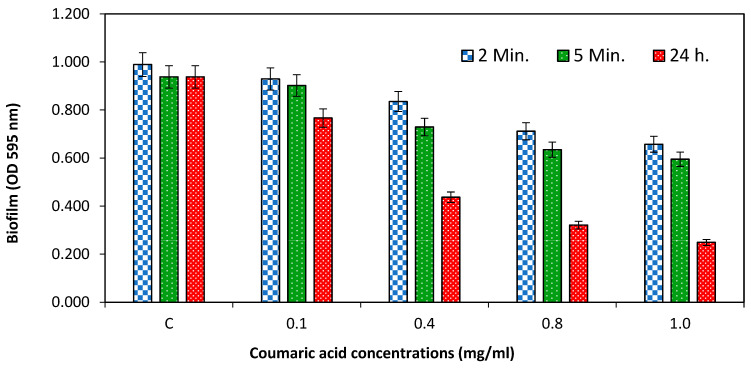
Potential of coumaric acid on the dispersion of *S. mutans* biofilm that had been pre-formed and treated at various time intervals.

To assess the impact of CA on pre-formed biofilms of *Streptococcus mutans*, we exposed the biofilm to various concentrations of CA and different exposure times (2 min, 5 min and 24 h). The results revealed that CA amounts ranging from 0.1 to 1 mg/mL had the ability to dislodge the 24 h old *Streptococcus mutans* biofilm (Figure 4). Most noteworthy, however, was biofilm dispersal (73.45%) at 1 mg/mL after 24 h of treatment. Also, as the amount of CA increased from 0.1 to 1 mg/mL, we noted a gradual dispersal of the pre-established bacterial biofilm. The ability to eradicate pre-formed biofilms is particularly notable at higher concentration (1 mg/mL) and could have practical implications for combating biofilm-related problems in various settings, such as healthcare and dentistry. Further research in this direction may unveil the full extent of CA’s effectiveness in biofilm eradication and it must be validated using various methods.

### 2.5. Effect on EPS (Polysaccharide and Protein)

**Figure 5:**
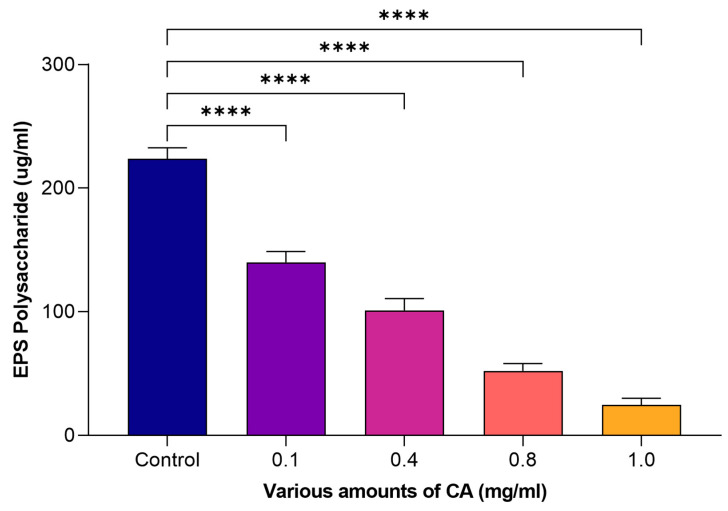
The efficacy of various amounts of CA on extracellular polysaccharide. The statistical tests used in these analyses were one-way ANNOVA followed by Tukey’s Multiple Comparisons Test. **** *p* ≤ 0.0001.

**Figure 6:**
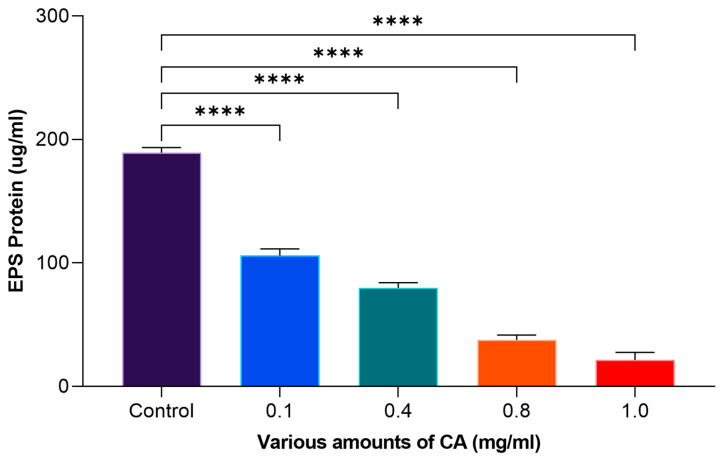
The efficacy of CA on extracellular protein. The statistical tests used in these analyses were one-way ANNOVA followed by Tukey’s Multiple Comparisons Test. **** *p* ≤ 0.0001.

Coumaric acid (CA) was evaluated at varying amounts, ranging from 0.1 to 1 mg/mL, to determine its efficacy in eliminating EPS, which includes polysaccharides and proteins. It was exhibited that (Figure 5), at 1 mg/mL of CA, the effects were more potent on the reduction in extracellular polysaccharides produced by *S. mutans*. In the control, there was 223.82 μg/mL of polysaccharide on the dentin surface, whereas the application of 1 mg/mL of CA reduced this amount to 24.80 μg/mL. The effect of CA on EPS elimination, specifically with regard to proteins, was also explored at varying concentrations. It was observed that 189.31 μg/mL of protein was observed in the control. However, at higher concentrations (1 mg/mL), CA significantly lowered the protein concentration to 21.47 μg/mL (Figure 6). Xu et al. [17] demonstrated the effect of CA to reduce extracellular polymeric substances (EPSs). They observed that CA has the potential to mitigate EPS. This reduction in extracellular proteins and polysaccharides suggested that CA has the efficacy to control the extracellular matrix of *S. mutans*, which is vital for its adhesion.

### 2.6. Microscopy of a Treated Biofilm

**Figure 7:**
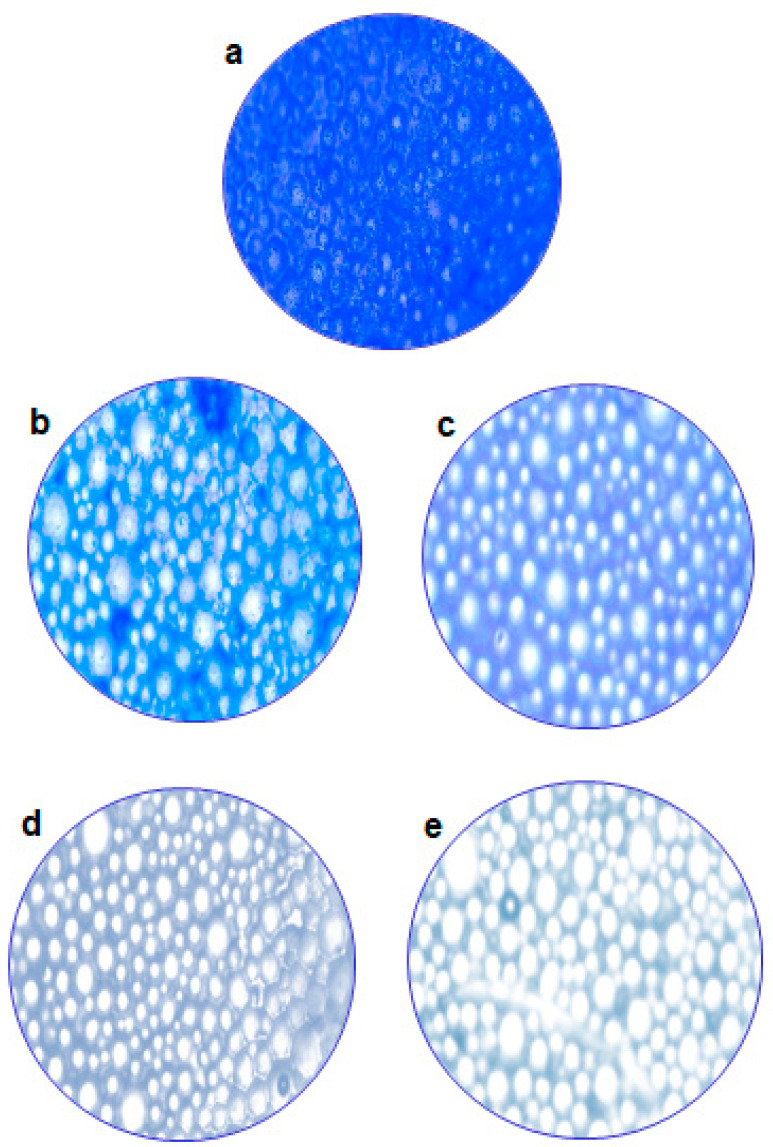
Microscopic examination of the effect of different coumaric acid concentrations on biofilm dispersal on the dentin surface. (**a**) control non-treated (**b**) 0.1 mg/mL (**c**) 0.4 mg/mL (**d**) 0.8 mg/mL (**e**) 1 mg/mL.

**Figure 8:**
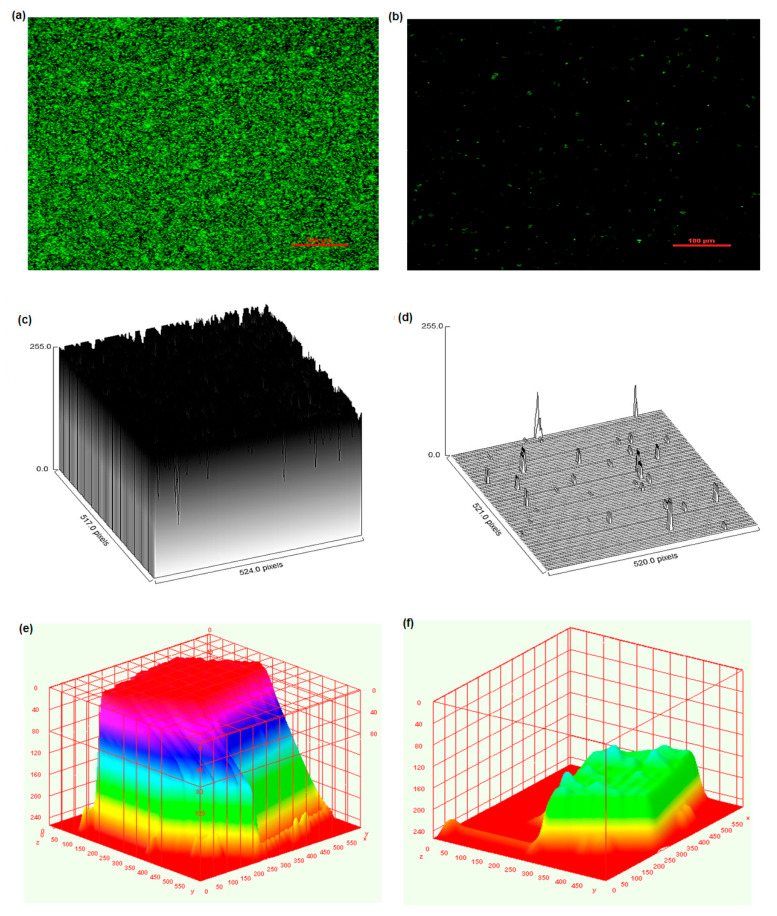
Florescence microscopic examination of effect of coumaric acid (1 mg/mL) on biofilm (**a**) florescence image of control non-treated biofilm, (**b**) florescence image of treated biofilm using 1 mg/mL CA, (**c**) surface plot of control biofilm, (**d**) surface plot of treated biofilm, (**e**) 3D surface interactive plot of control biofilm, (**f**) 3D surface interactive plot of treated biofilm.

To provide additional validation of our results, we conducted a closer evaluation of biofilm control using both light and fluorescence microscopes. Figure 7a represents a microscopic image of the control (untreated) biofilm. In Figure 7e, it is observed that at 1 mg/mL of CA, less biofilm was present compared to the other tested concentrations. Al-Juboori and Yusaf [24] studied and demonstrated that light microscopy is one of the simplest techniques for the analysis of biofilms. ImageJ software (version 1.54 g) was used to process florescence images (Figure 8a,b), and it was observed that the biofilm mass was 213 μm^2^ at 1 mg/mL of CA, and the percent surface coverage was 0.079%, while for the control biofilm, the mass was 254,113 μm^2^ and percent surface coverage was 93.80%. Furthermore, the 3D surface plots (Figure 8c–f) showed that the biofilm treated with CA displayed more wide-spread, less dense and less fibril-like projections compared to the non-treated biofilm. The results suggest that CA can mitigate the biofilm caused by *S. mutans*, especially at higher amounts. The decrease in biofilm mass and surface coverage along with the structural difference in the treated and control samples demonstrated the potential of CA as a promising agent for biofilm mitigation. CA demonstrated antibiofilm activity against cariogenic bacteria. *S. mutans* suggests potential applications in the development of antibiofilm dental formulas for therapeutic use or oral hygiene practices. Future studies should also explore the toxicity of CA to ensure its safety in various applications.

### 2.7. Interaction of CA with Structure of S. mutans 2I2J

**Figure 9:**
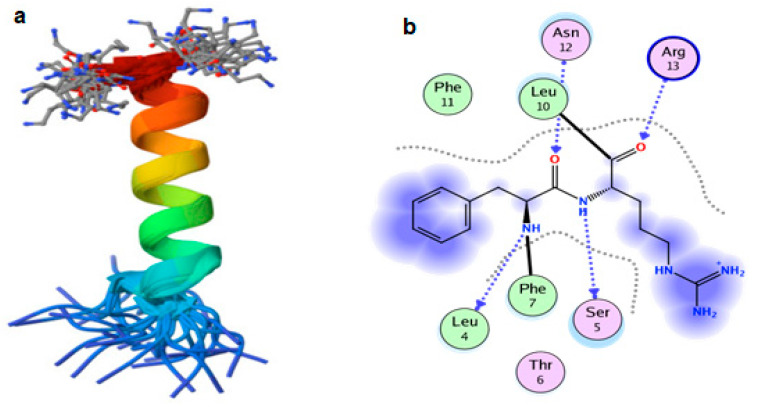
(**a**) The 3D structure of 2I2J and (**b**) 2D interaction of 2I2J and CA.

Several studies applied molecular docking tools to confirm the activity of natural compounds, discover new ones or design pharmacological compounds for the treatment of numerous diseases [25,26]. The results showed that the ligand (coumaric acid) studied has a comparable position and orientation inside the binding site of the Competence Stimulating Peptide (CSP) UA159sp (PDB ID: 2I2J) (Figure 9a). Furthermore, the affinity of any small molecule can be thought of as a unique instrument in the realm of drug design. The affinity of organic molecules and the free energy of binding have a relationship. This relationship can help anticipate and interpret the activity of organic chemicals towards given target proteins. The selected compound (CA) had favorable binding free energies value in negative Kcal/mol (E score), as indicated in Table 1. The proposed binding mode of coumaric acid revealed an affinity value of −4.29 towards the 2I2J peptide. The obtained results of molecular docking interactions (Figure 9b) reflected a low energy score with 2I2J. In the study of the binding properties of coumaric acid, N 1, N 21, O 4, O 24 atoms form hydrogen bond contact with LEU4, SER5, ASN12 and ARG13 amino acid residues of *Streptococcus mutans* 2i2J protein, respectively. These results suggest that CSP 2i2J could be one of the targets for CA to mitigate *S. mutans* biofilm formation.

The toxicity prediction revealed that CA is non-toxic regarding its tumorigenic, irritant and mutagenic effects. However, risk alerts via Property Explorer Applet are not intended to serve as entirely reliable toxicity predictions, and the absence of risk alerts does not imply that a substance is entirely free of any toxic effects. In a study performed by Devi et al. [27], CA demonstrated safety in acute and sub-acute toxicity tests on mice, showing no adverse effects on vital organs at the tested doses, indicating its potential as a safe agent.

## 3. Materials and Methods

### 3.1. Microbe, Chemicals and Accessories

Formerly isolated and identified dental plaque bacteria, *Streptococcus mutans*, were used for growth and biofilm studies. All chemicals were purchased from Sigma Aldrich, Deajunge, Republic of Korea, Alfa Aesar (Ward Hill, MA, USA). Glassware and accessories were purchased from MUSAJI Adam and Sons (Abbottabad, Pakistan), and polystyrene microplates (24 wells), glass slides and dentin were purchased from local suppliers.

### 3.2. Cultivation of Bacteria

For cultivation of *Streptococcus mutans*, Nutrient broth (NB) was utilized. Bacteria were inoculated in sterilized NB under aseptic conditions. Then, culture was incubated at 37 °C with shaking at 120 rpm for a duration of 24 h [5,28].

### 3.3. Bacterial Growth Assays

The first round of testing aimed to evaluate the influence of coumaric acid (CA) on planktonic bacterial growth. Bacterial inoculation was carried out in NB, and the bacterial samples were cultured at 37 °C with shaking at 200 rpm for a duration of 24 h. After that, microtiter plates were inoculated with the bacterial cultures at optical density of 0.001 (≈to 1.12 × 10^6^ CFU/mL). Next, 50 μL of CA (with concentrations ranging from 0.01 to 1 mg/mL) was added into each well from stocks of CA. After that, NB was added in each well. The bacterial microtiter plates were then incubated at 37 °C in a shaking incubator at 120 rpm for 24 h. Equivalent to the treated group, a control group was included in the experiment. The main difference was that, in the control group, an equivalent volume of distilled water (sterile) was used instead of CA. The absorbance of the bacterial cells was quantified at 600 nm using spectrophotometer. Each experiment was conducted in triplicate [29].

### 3.4. Biofilm Formation Control in Microtiter Plate

Fifty microliters of the CA solution from various stocks of CA was added to each well of a (24-well) microtiter plate [30] to achieve desired CA concentrations in the wells ranging from 0.01 to 1 mg/mL. Subsequently, 20 μL of bacterial culture was added to each well, and 930 μL of NB was added to each well to achieve a total volume of 1 mL. Afterward, plates were incubated for 24 h in a shaking incubator set at 120 rpm and 37 °C. After incubation, media were removed from each well, and plates were carefully rinsed with sterilized distilled water to minimize any loss of the biofilm. Plates were air-dried. After this, 1 mL of 99.9% ethanol was added, and plates were kept at room temperature for 15 min. Then, ethanol was removed, and the plates were air-dried. Each well was stained with 1 mL of crystal violet solution (0.1% *w*/*v*), and the plates were incubated for 20 min at room temperature. After staining, plates were washed to remove excess dye, and plates were air-dried. The dye bound to the biofilm was re-dissolved in 1 mL glacial acetic acid solution (33% *v*/*v*). Then, absorbance of the crystal violet dye was quantified at 595 nm using spectrophotometer [31]. Each experiment was performed in triplicate.

### 3.5. MTT Biofilm Viability Assay

The MTT test, a widely recognized viability assessment test, was employed for checking the influence of CA on *Streptococcus mutans* cell viability in biofilms. In the biofilm formation assay, various amounts of CA were introduced into each well, as outlined in Section 3.4. After the biofilm formation, the biofilms were gently rinsed with sterile distilled water. Following rinsing, 100 μL of MTT (5 mg/mL stock) and 900 μL of phosphate-buffered saline (PBS) were added to each well of the microtiter plates, and plates were incubated at 37 °C for 3 h. The reduction reaction of MTT led to the formation of formazan crystals, which were then dissolved in dimethyl sulfoxide (DMSO). The absorbance of the samples was recorded at 570 nm via spectrophotometer.

### 3.6. Biofilm Eradication Studies in Polystyrene Plates

We also evaluated the influence of varying CA amounts on the eradication of pre-existing biofilms. For this purpose, biofilms were cultured in NB in 24-well microtiter plates for 24 h at 37 °C under shaking conditions at 120 rpm. Subsequently, the biofilms were gently washed with sterilized PBS. Various amounts of CA (ranging from 0.1 to 1 mg/mL) were incorporated into each well of the plate. Concurrently, a control group without CA was exposed to the same testing conditions. After that, plates were incubated for 2 min, 5 min and 24 h in an incubator on shaker, keeping a speed of 100 rpm at 37 °C [29,32]. Biofilms were analyzed via crystal violet assay, as mentioned in Section 3.4.

### 3.7. Biofilm Assays on Glass Slides Coated with Dentin

Bacteria were cultivated in NB, and agitation was performed at 120 rpm in a shaking incubator. The *S. mutans* cells on the plates were properly diluted to achieve the needed concentrations (0.001 OD). The bacterial cultures were then transferred to fifty-milliliter tubes, and tubes were vortexed. Then, slides were kept in 50 mL falcon tubes. Desired concentrations of CA were added to each tube from different stock cultures to achieve desired CA concentrations from 0.1 to 1 mg/mL. After that, tubes were incubated at 37 °C under shaking conditions for 24 h at 120 rpm. In the control tube, CA was replaced with sterilized distilled water [32]. Slides were subjected to microscopy and EPS analysis.

### 3.8. Extraction of EPS

EPS attached to the dentin surfaces was extracted. For this, a cell scraper was employed to remove the biofilm from the dentin surface, after which extracted mass was vortexed and dissolved in 20 mL of PBS in 50 mL falcon tubes. The biofilm samples were vortexed for five minutes within the same falcon tube. After this step, the samples were centrifuged at 5000 rpm for twenty minutes. After centrifugation, the supernatant, which contained the soluble EPS, was shifted to new tube, while the residual pellet was specified as the crude biomass [33].

### 3.9. Polysaccharides and Protein Quantification

One milliliter of the EPS solution was gently added into a fifteen-milliliter tube, which was then labeled. After this, 0.5 mL of a 5% phenol solution was added to the tube. Then, 2.5 mL of concentrated H_2_SO_4_ was added to the mixture. Complete mixing was carried out, and the blend kept incubating for 10 min at room temperature. Then, 1 mL of the sample was taken in cuvette, and the absorbance was assessed using a spectrophotometer at 492 nm [34]. For quantification of extracellular proteins, Bradford assay was performed [35].

### 3.10. Microscopy of a Treated Biofilm

Initially, simple microscopy was employed for visualization of biofilm samples. For this, 1 mL of crystal violet solution (0.1%) was added on each slide, and it was incubated for 15 min at room temperature. After staining, the slides were gently washed with sterilized water. Slides were visualized under light microscope using objective lens of 40× [36], and images were captured using digital camera.

Slides containing biofilm samples were also subjected to fluorescence microscopy. Briefly, samples were washed with saline water and stained with fluorescein isothiocyanate (FITC) for 20 min in the dark. After staining, the unbound stain was removed with sterilized water. Stained samples were viewed, and images were obtained at 530 nm emission and 488 nm excitation. Images were processed for surface coverage (%) and biofilm biomass (μm^2^) using ImageJ Software (version 1.54 g) [37]. Then, 3D surface plots were constructed from the green florescence intensity of the samples via ImageJ 3D Surface plots plugin.

### 3.11. Experimental Methods of Molecular Docking Interaction and Toxicity Analysis

Molecular operating environment (MOE) software (2015.10) was used to conduct molecular docking investigations on possible interaction pattern of CA with Competence Stimulating Peptide (UA159sp, PDB ID: 2I2J) of *S. mutans*. Protein (2I2J) 3D structure in PDB format was downloaded from the Protein Data Bank (PDB), and structure of CA (CID 637542) was downloaded from PubChem. At first, the protein file was prepped using the built-in function. Then, water molecules and hetero molecules were manually deleted from the downloaded proteins, and polar hydrogen molecules were added for interaction. Then, the best sites for interaction were found by computing, the largest site was selected for interaction and the docking site was specified as dummy atoms. Two poses were selected for docking. The library of compound was browsed, CA was loaded and prepped into MOE database file and dock calculations were run automatically. At the end of the docking processes, the obtained poses were carefully studied, and the ones having the best ligand–protein interaction were selected. Furthermore, we predicted possible toxicity of CA via OSRIRIS Property Explorer Applet [38].

### 3.12. Statistical Analysis

Each test was performed three times. Microsoft Excel (Office 365) was used to determine the average and standard error (S.E.). S.E. was represented as error bars in the graphs using Microsoft Excel and GraphPad Prism (9.5.1). To evaluate the significance of the results, a two-tailed Student’s *t*-test was employed, with the significance level set at *p* < 0.05. The performance of this statistical test was carried out using Microsoft Excel.

## 4. Conclusions

CA exhibited its efficacy in combating the biofilm formed by *Streptococcus mutans* associated with dental plaque. Especially, at higher amounts, CA displayed a noticeable inhibition in planktonic bacterial growth. Furthermore, CA exhibited the ability to prevent the formation of biofilms on dentin surfaces. The MTT assay further showed that CA could reduce the viability of bacteria within biofilms, with an obvious decrease in cell viability as the amount of CA increased. CA also demonstrated its efficacy for dispersing or eradicating pre-existing biofilms on dentin surfaces, featuring its biofilm-dispersing abilities. Likewise, biomass analysis, as well as light and fluorescence microscopy, provided additional insights for confirmation of CA’s biofilm-mitigating properties. The reduction in extracellular polymeric substances (EPSs), involving proteins and polysaccharides, on dentin surfaces was associated to the mitigation of biofilms by CA. The analysis of the untreated biofilm exhibited a dense structure with abundant fibril-like projections, as depicted in 3D surface plots. Moreover, molecular docking predicted a possible interaction pattern between CA and the Competence Stimulating Peptide (2I2J). This dual approach, combining microscopic observations with molecular insights, increased our understanding of biofilm complexity, and it provides vital information for future research.

## Figures and Tables

**Table 1:** Interaction of CA with structure of *S. mutans* 2I2J.

Ligand	Receptor	Interaction	Distance	E (kcal/mol)
N 1	LEU 4	H-donar	2.75	−6.3
N 21	SER 5	H-donar	2.76	−6.2
O 4	ASN 12	H-acceptor	2.87	−6.1
O 24	ARG 13	H-Acceptor	2.80	−5.3

## Data Availability

The data presented in this study are available in article.

## Abbreviations

CA	Coumaric acid
MTT	3-(4,5-dimethylthiazol-2-yl)-2,5-diphenyl-2H-tetrazolium bromide
EPS	Extracellular Polymeric Substances
CSP	Competence Stimulating Peptide
QS	Quorum Sensing
MIC	Minimum Inhibitory Concentration
OD	Optical Density
LEU	Leucine
SER	Serine
ASN	Asparagine
ARG	Arginine
NB	Nutrient Broth
PBS	Phosphate Buffer Saline
DMSO	Dimethyl Sulfoxide
FITC	Fluorescein Isothiocyanate
PDB	Protein Data Bank
